# Prenatal risk assessment of Xp21.1 duplication involving the *DMD* gene by optical genome mapping

**DOI:** 10.26508/lsa.202402780

**Published:** 2024-08-08

**Authors:** Yuanyuan Zhang, Qiang Du, Haiming Gao, Yujie Pan, Ningyang Liu, Chuang Qiu, Xiaoliang Liu

**Affiliations:** 1 Department of Clinical Genetics, Shengjing Hospital of China Medical University, Shenyang, China; 2 Center of Reproductive Medicine, Shengjing Hospital of China Medical University, Shenyang, China; 3 Department of Laboratory Medicine, Shengjing Hospital of China Medical University, Shenyang, China; 4 Department of Orthopedics, Shengjing Hospital of China Medical University, Shenyang, China; 5 Key Laboratory of Reproductive Health, Liaoning Research Institute of Reproductive Health and Development, Reproductive Hospital of China Medical University, Shenyang, China

## Abstract

Structural variants (SVs) of unknown significance are great challenges for prenatal risk assessment, especially when involving dose-sensitive genes such as *DMD*. The pathogenicities of 5′-terminal *DMD* duplications in the database remain controversial. Four prenatal cases with Xp21.1 duplications were identified by routine prenatal genomic testing, encompassing the 5′-UTR to exons 1–2 in family 1 and family 2, and to exons 1–9 in family 3. The duplication in family 4 was non-contiguous covering the 5′-UTR to exon 1 and exons 3–7. All were traced to unaffected males in the family pedigrees. A new genome-wide approach of optical genome mapping was performed in families 1, 2, and 3 to delineate the breakpoints and orientation of the duplicated fragments. The extra copies were tandemly inserted into the upstream of *DMD*, preserving the integrity of ORF from the second copy. The pathogenicities were thus reclassified as likely benign. Our data highlight the importance of structural delineation by optical genome mapping in prenatal risk assessment of incidentally identified SVs involving *DMD* and other similar large dose-sensitive genes.

## Abstract (precis)

It classifies the Xp21.1 duplications involving 5′-terminal *DMD* in prenatal case series as likely benign using OGM, which emphasizes the importance of structural evaluation on SVs involving *DMD* and other large dose-sensitive genes.

## Introduction

Genomic structural variants (SVs) are common genetic causes of birth defects, including aneuploidies, inversions, translocations, and copy-number variations (CNVs). With the extensive application of prenatal genomic testing, some incidental findings that are inconsistent with the detection indicators may be observed. These variants of uncertain significance (VUS) arouse great maternal anxiety and make the pregnancies problematic for genetic counseling. Being the largest known gene in human, the dystrophin (*DMD*) gene spans ∼2.4 Mb on chromosome Xp21.2–p21.1 and consists of 79 exons in the muscular transcript (Den Dunnen et al, 1992). Loss-of-function variants in *DMD* cause Duchenne muscular dystrophy (DMD) (OMIM 310200), typically presenting as a progressive disorder in young boys affecting both skeletal and cardiac muscles (Darras et al, 2000). Specific in-frame variants in the *DMD* gene cause Becker muscular dystrophy (BMD) (OMIM 300376), which has a later onset and a milder and varied clinical course (Darras et al, 2000). Most carrier females do not manifest any symptoms or abnormalities, whereas some may have isolated elevated serum creatine kinase (CK) levels (Matthews et al, 1995; Prior & Bridgeman, 2005; Ishizaki et al, 2018; Zhong et al, 2019; Liu et al, 2023; Sarkozy et al, 2023). The most common pathogenic *DMD* variants are deletions (65–70%) and duplications (∼11%) of one or more exons (Bladen et al, 2015). However, not all the *DMD* duplications predispose to muscular dystrophy, even those reported as pathogenic in the public database (Aartsma-Rus & den Dunnen, 2019). It is challenging to interpret the pathogenicities and predict the phenotypic consequences, especially for male fetuses with incidental findings of CNVs involving *DMD* duplications. Detailed evaluation of structural arrangement and reading-frame integrity is needed to enrich our experience in prenatal risk assessment.

Optical genome mapping (OGM) is a next-generation cytogenetic technology that is capable of detecting all types of genome-wide chromosomal abnormalities including triploidies, aneuploidies, >500-bp CNV, >70-kb balanced chromosomal structural rearrangement, >25-Mb loss of heterozygosity (LOH), and mosaicisms (Dremsek et al, 2021; Mantere et al, 2021). In addition, it holds the potential to refine the localization of breakpoints and determine the orientation of repeated/inserted fragments (Sahajpal et al, 2023). Growing evidence has been accumulated about the usage of OGM in postnatal analysis of constitutional genetic disorders and hematological malignancies (Mantere et al, 2021; Neveling et al, 2021). Nevertheless, studies on its application in prenatal diagnostic settings remain limited.

Herein, four prenatal cases with incidentally identified Xp21.1 duplications involving 5′-terminal partial exons of the *DMD* gene were reported. Three of them underwent an OGM assay, each showing a tandemly duplicated fragment and an intact *DMD* copy. The pathogenicities were reclassified from VUS to likely benign, which highlighted the great value of prenatal application of OGM.

## Results

### Prenatal incidental finding of Xp21.1 duplications by whole-exome sequencing (WES) and CNV sequencing (CNV-seq)

**Figure 1.**
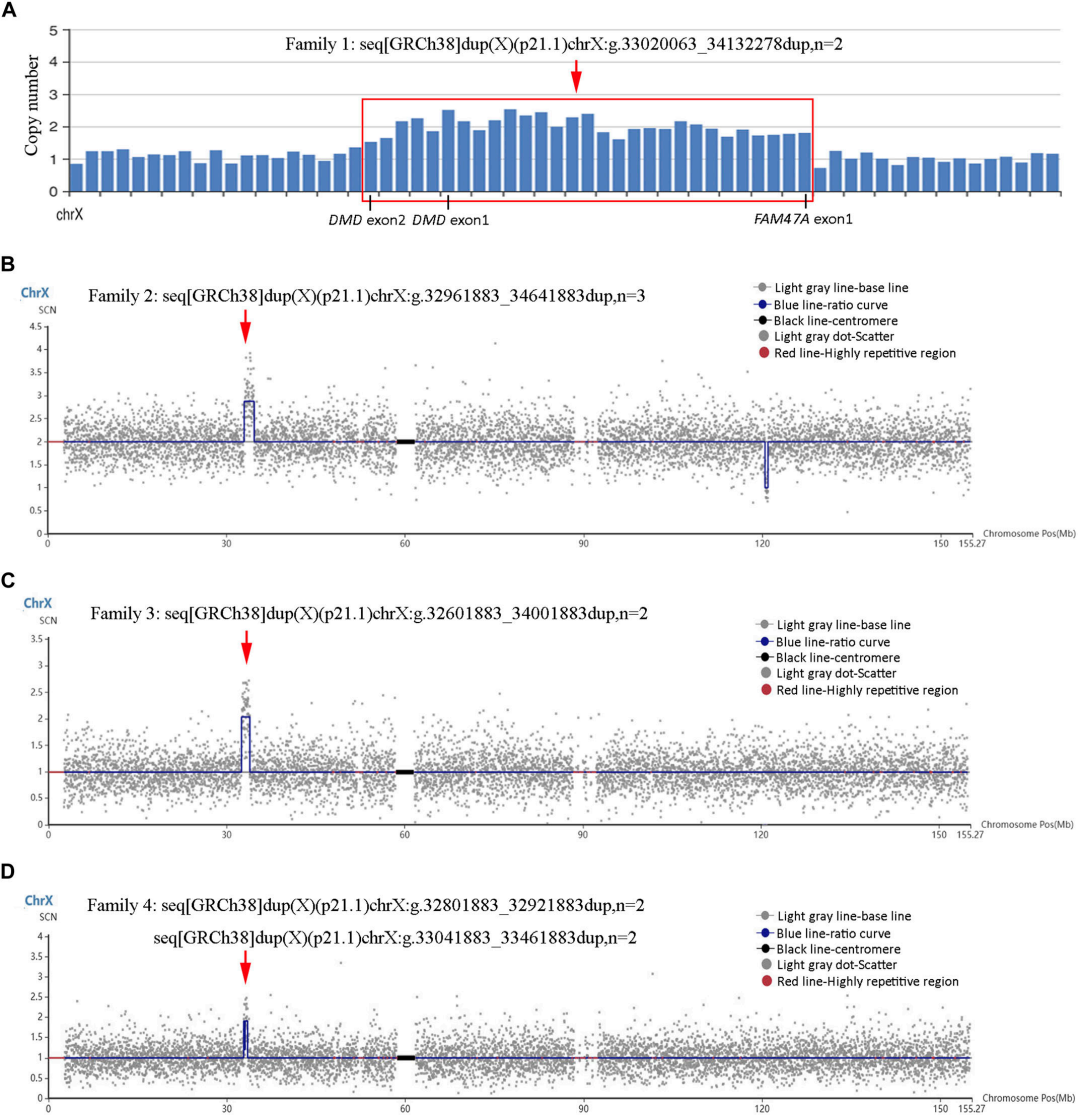
Xp21.1 duplications identified by routine prenatal genomic testing. **(A, B, C, D)** Xp21.1 duplications identified in the fetuses from family 1 by whole-exome sequencing (A) and from families 2, 3, and 4 by copy-number variation sequencing (B, C, D). Duplicated regions are indicated by the red frame and red arrows.

The pregnant woman from family 1 was 32-yr-old gravida 1, para 0 who underwent amniocentesis for unilateral cheilopalatognathus on the fetus at 23 wk of gestation. No genetic etiology was identified related to cheilopalatognathus by WES. However, a 1.11-Mb duplication in Xp21.1 (GRCh38:chrX:g.33020063_34132278, n = 2) was found in the male fetus (Fig 1A). The duplication encompassed the 5′-UTR and exons 1–2 of the *DMD* gene according to the WES data.

The pregnant woman from family 2 was 26-yr-old gravida 1, para 0 who underwent amniocentesis at 20 wk of gestation for fetal pulmonary cystadenoma. A 1.68-Mb duplication in Xp21.1 (GRCh38:chrX:g.32961883_34641883, n = 3) was detected in the female fetus by CNV-seq on amniocytes (Fig 1B). The duplicated fragment overlapped the 5′-UTR and exons 1–2 of the *DMD* gene.

The pregnant woman from family 3 was 33-yr-old gravida 2, para 0 who underwent amniocentesis at 19 wk of gestation for a previous pregnancy of trisomy 21. A 1.4-Mb duplication in Xp21.1 (GRCh38:chrX:g.32601883_34001883, n = 2), encompassing the 5′-UTR and exons 1–12 of *DMD*, was detected by CNV-seq in the male fetus (Fig 1C).

The pregnant woman from family 4 was 37-yr-old gravida 3, para 0 who underwent amniocentesis at 19 wk of pregnancy because of advanced maternal age. Two non-contiguous duplications in Xp21.1 (GRCh38:chrX:g.32801883_32921883 and g.33041883-33461883, n = 2), with the sizes of 0.12 and 0.42 Mb, respectively, were found by CNV-seq in the male fetus (Fig 1D). The duplication involved the 5′-UTR and exon 1 and exons 3–7 of *DMD*.

### Pedigree tracing by multiplex ligation-dependent probe amplification (MLPA) analysis

**Figure 2.**
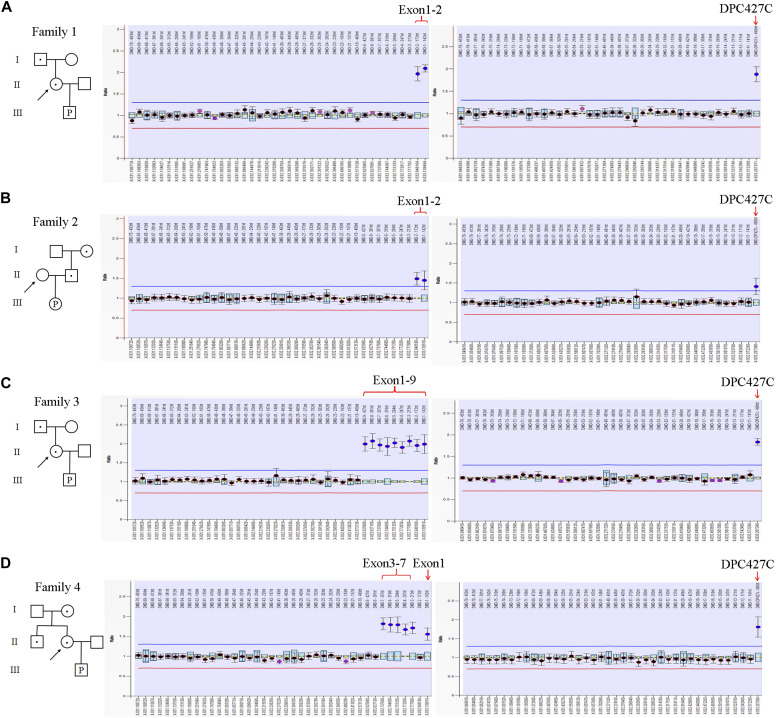
Pedigree analysis by multiplex ligation-dependent probe amplification (MLPA). **(A, B, C, D)** Pedigrees and representative data of MLPA in families 1, 2, 3, and 4 (A, B, C, D). The white square or circle with a black dot represents an asymptomatic carrier. The white square or circle with a “P” inside represents the fetus. The mothers of the fetuses are indicated by black arrows. The x-axis represents MLPA probes of P034 and P035, and the y-axis represents the probe dosage quotient. The duplicated regions above the blue line (probe dosage quotient of 1.35) are indicated by red braces and arrows.

MLPA was performed on the one hand to confirm the duplications involving *DMD*, and on the other hand to figure out the pedigree tracing. The duplication in the fetus of family 1 involved probes of DP427C (5′-UTR) and exons 1–2 of *DMD*, which was inherited from the mother and from the asymptomatic maternal grandfather (Fig 2A). The duplication in the fetus of family 2 also involved the probes of DP427C and exons 1–2, which was paternally inherited, and the father was physically healthy (Fig 2B). The duplication in the fetus of family 3 involved probes of DP427C and exons 1–9 of *DMD* by MLPA, which was indeed inconsistent with the CNV-seq data (from the 5′-UTR to exons 1–12). It was maternally inherited and was further traced to the asymptomatic maternal grandfather (Fig 2C). The non-contiguous duplication involved probes of DP427C, exon 1, and exons 3–7 in the fetus of family 4, which was maternally inherited. It was traced to the maternal grandmother and the maternal uncle who was physically healthy (Fig 2D).

### Structural delineation by OGM analysis

**Figure 3.**
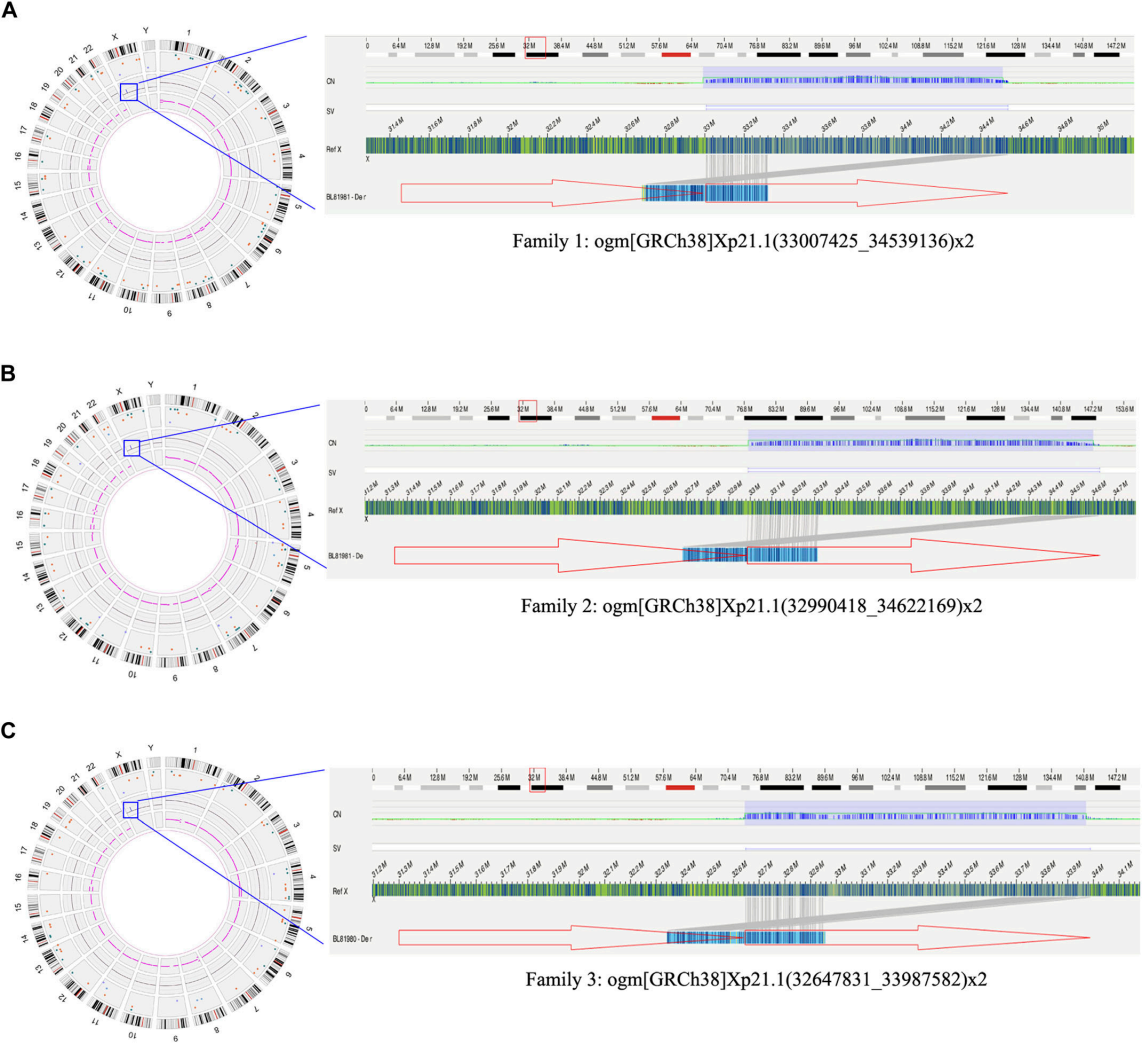
Optical genome mapping results. **(A, B, C)** Structural delineation of the Xp21.1 duplications by optical genome mapping in adult male carriers of families 1, 2, and 3 (A, B, C). The circos plots are shown on the left, with the copy-number gains on Xp21.1 framed with blue boxes. The amplified genomic browser views of the Xp21.1 duplications are shown on the right. The alignments of the sample’s consensus map (blue bar) with the reference consensus map (green bar) illustrate duplications of 1.53 Mb, 1.63 Mb, and 1.34 Mb, respectively, all of which are tandemly duplicated (indicated by the red arrows and gray lines).

The pregnant women in families 1, 2, and 3 requested for further eliminating risks. Thus, the adult male carriers in these families underwent OGM analysis to further delineate the structural arrangement and exclude reading-frame disruption. As shown in the circos plots of Fig 3A–C, 1.53-, 1.63-, and 1.34-Mb copy-number gains (n = 2) in Xp21.1 were found in the three families, respectively. In comparison with the human reference genome GRCh38, the SVs could be described as ogm[GRCh38]Xp21.1(33007425_34539136)x2 for family 1, ogm[GRCh38]Xp21.1(32990418_34622169)x2 for family 2, and ogm[GRCh38]Xp21.1(32647831_33987582)x2 for family 3 according to the International System for Human Cytogenomic Nomenclature (ISCN) (Moore et al, 2023). The genome map against the reference showed the extra copies were tandemly inserted into the upstream of *DMD*, leaving the downstream *DMD* copy intact. The extra fragments were supposed to be non-functionally influencing through either nonsense-mediated mRNA decay or extremely short protein truncates.

### Pregnancy outcomes and follow-up

All the adult males with Xp21.1 duplications in the four families were persuaded to check CK levels, returning with values within normal ranges (data not shown). Based on OGM data and family co-segregation analyses, the Xp21.1 duplications in families 1, 2, and 3 were reclassified as likely benign according to the American College of Medical Genetics and Genomics (ACMG) guideline for CNVs, using the evidence of “1A (0 points), 2J (0 points), 3A (0 points), 4J (−0.6 points), and 5B (−0.3 points)” (Riggs et al, 2020). The pregnancies, including those in family 4, were suggested to be continued considering the reduced risk of pathogenicities. By the time of the last follow-up, all the fetuses had been born. The babies in families 1, 2, and 3 presented normally with ages of 7, 18, and 5 mo, respectively. The boy aged 2 yr in family 4 was also asymptomatic of neuromuscular disorders. CK levels had also been determined on the babies in family 2 and family 4, with results showing normal. The rest two families denied CK testing for the babies without any indications.

### Review of the public database

**Figure 4.**
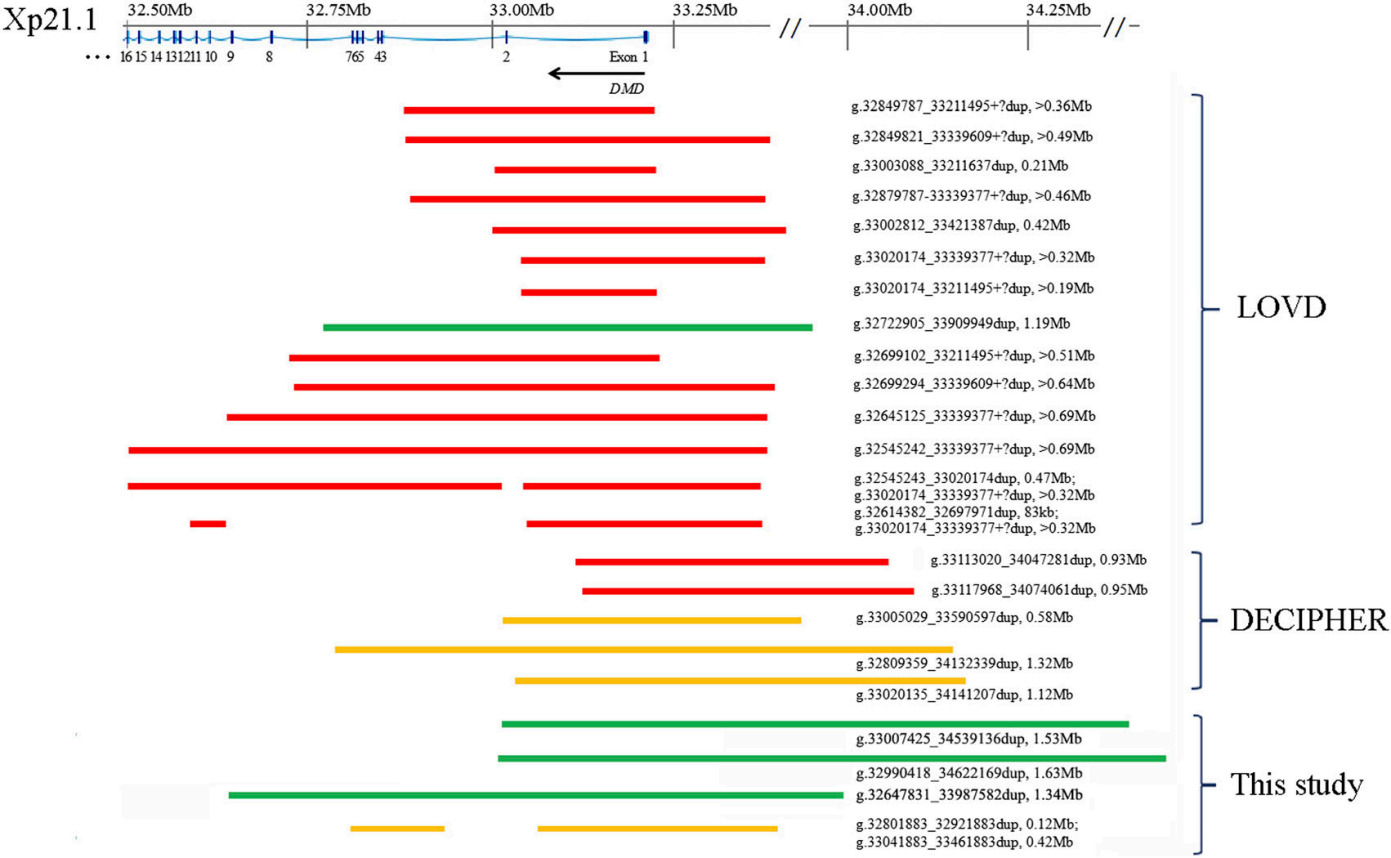
Schematic representation of the Xp21.1 duplications. Schematic representation of the Xp21.1 duplications involving 5′-terminal *DMD* in public databases and in the present study. The duplicated regions (reference genome GRCh38) are depicted as varied lengths of bars, with the ranking of pathogenicity indicated by colors (red as pathogenic/likely pathogenic, yellow as variants of uncertain significance, and green as benign/likely benign). LOVD, Leiden Open Variation Database; DECIPHER, Database of genomic variation and Phenotype in Humans using Ensemble Resources.

In Leiden Open Variation Database (LOVD), 14 duplications covering 5′-terminal exons of *DMD* have been recorded (Fig 4), including exon 1, exons 1–2, exons 1–7, exons 1–9, exons 1–16, and two non-contiguous duplications involving exons 1, 3–16, and exons 1, 10–11, respectively. Thirteen of them were recorded as pathogenic/likely pathogenic, and only one with exons 1–7 was ranked as likely benign. As in the Database of genomic variation and Phenotype in Humans using Ensemble Resources (DECIPHER), 5 duplications covering 5′-terminal *DMD* could be found. The two extending from the 5′-UTR to exon 1 were pathogenic, and the rest three encompassing the 5′-UTR to exons 1–2 or exons 1–7 were VUS. By adding our case series, we expanded the duplication profile and refreshed the pathogenicities with structural delineation (Fig 4).

## Discussion

In this study, prenatal case series with Xp21.1 duplications of uncertain significance involving 5′-terminal partial exons of *DMD* were evaluated by OGM. The duplications were reclassified as likely benign after structural delineation. Our data support the high efficacy of OGM in prenatal application on tricky SVs involving dose-sensitive genes.

DMD and BMD are the most common neuromuscular disorders, and nearly one third of the cases are de novo (Bladen et al, 2015). Prenatal diagnosis has been typically performed in pregnancies with ascertained family histories, whereas de novo cases are generally ignored before delivery. Being the largest known human gene, SVs involving the complete or partial *DMD* gene are recognizable and occasionally reported at the intrauterine stage with the wide application of genomic prenatal testing, such as non-invasive prenatal screening (NIPS), chromosome microarray analysis (CMA), CNV-seq, and WES (Brison et al, 2019; Hiraide et al, 2019; Shi et al, 2021). However, duplications are intractable for prenatal risk assessment because of not only the non-penetrant fetal phenotype and limited time and family members for pedigree tracing, but also the uncertainty of insertion position and orientation of the extra copies. A recent carrier screening for *DMD* by MLPA on 85,737 general population revealed 80 cases of VUS, including 58 duplications of the DP427C promoter and 11 duplications involving N-terminal exons, which were defined as “difficult to predict” by public databases (Singer et al, 2023). Therefore, the effects of the 5′-terminal *DMD* duplication on the reading frame are hard to, but necessary to, be dissected.

To the best of our knowledge, no literature about prenatal structural evaluation on Xp21.1 duplications involving the 5′-UTR and/or 5′-terminal partial exons of *DMD* has been published. In comparison with the duplications of similar regions and sizes in LOVD, 11 records were determined by MLPA and three by next-generation sequencing. All were ranked as pathogenic/likely pathogenic except for one as likely benign based on the familial co-segregation analysis. In DECIPHER, three of the five records with Xp21.1 duplication encompassing the 5′-UTR and 5′-terminal partial exons of *DMD* were ranked as VUS, and the rest two were ranked as likely pathogenic. None of these records were provided with detailed structural or sequential information. Three duplications in our case series were deciphered by OGM to be tandemly inserted into the upstream 5′-UTR. The first copy will result in a frameshift, and the ORF will be preserved from the second copy inserted. Combined with the family histories, we reassessed these duplications as likely benign. Likewise, long-read whole-genome sequencing has been reported in two single prenatal cases with Xp21.1 duplications involving 3′ partial *DMD* exons (Chin et al, 2021; He et al, 2022). The duplication encompassing the 3′-UTR and exons 62–79 of *DMD* (chrX:g.30939526_31362638) in a male fetus was confirmed as a direct repeat inserted downstream of *DMD*, and was co-segregated with a healthy maternal uncle. The duplication in the other male fetus was a complex non-contiguous fragment containing two copies of exons 51–53, and one copy of exons 64–79 and intron 55 inserted outside of the *DMD* gene region. The complex structural rearrangement was inherited from the mother and the asymptomatic maternal grandfather. Taken together, duplications involving 5′- or 3′-terminus are likely to preserve an intact *DMD* copy. Without solid structural delineation, we should be very prudent to consider pathogenic/likely pathogenic variants when encountering such CNVs during regular prenatal screening.

By reviewing literature, complex rearrangements involving the *DMD* gene including double deletions or non-contiguous duplications were rarely reported (Hoop et al, 1994; Fenollar-Cortés et al, 2008; del Gaudio et al, 2008; Zimowski et al, 2013; Kumar et al, 2020; He et al, 2022). The molecular mechanisms underlying such copy-number gains and loss have been proposed to be non-homologous end-joining (NHEJ) repair, homologous recombination, and errors of DNA replication (del Gaudio et al, 2008). Most of them were reported on DMD/BMD patients with intragenic exons affected. They were presumed to be pathogenic without detailed in-depth analysis of structural delineation or transcription. One exception was about a female patient diagnosed with hereditary neuropathy with liability to pressure palsies (HNPP, caused by 17p12 microdeletion) who was incidentally found having complex non-contiguous fragments involving 3′-terminal partial exons of *DMD*. Delineated by long-read and Sanger sequencing, the complex fragment was inserted outside *DMD* and was reclassified as likely benign (He et al, 2022). Our case 4 has non-contiguous duplications involving the 5′-UTR, and exons 1 and 3–7, which were traced to unaffected maternal uncle. It is a pity that OGM was refused by the family. The boy aged 2 yr is asymptomatic at present with the normal CK level. A high possibility is that the rearrangement copy may be inserted into the upstream 5′-UTR or elsewhere outside the *DMD* gene region, retaining an intact *DMD* ORF. Long-term follow-up will be performed on our case 4.

Complex SVs are always insoluble for conventional genetic technologies. The new OGM technology is capable of whole-genome imaging by de novo assembly of labeled and linearized ultra-high molecular weight DNA (Goumy et al, 2023). It is currently a promising diagnostic tool for constitutional chromosomal aberration with great superiority over karyotyping for high resolution, over FISH for high throughput, and over CMA/CNV-seq/WES for detection of balanced/imbalanced chromosomal rearrangements with informed localization and orientation of the fragments (Sahajpal et al, 2021; Goumy et al, 2023). Long-read sequencing, also known as third-generation sequencing (TGS), could depict the SVs and define the precise breakpoints (Geng et al, 2021). However, the expensive price handicapped its wide utility. Taking advantage of the OGM technology, we pioneered the usage in prenatal assessment for SVs involving *DMD* duplications. Comparatively, it is rational to see the duplicated fragment determined by OGM in family 1 was larger than that by WES. The breakpoints suggested by OGM in families 2 and 3 were in about 20- to 40-kb proximity to those determined by CNV-seq. It is noteworthy that the downstream breakpoints at intron 9 by OGM in family 3 were in accordance with the data of MLPA but not CNV-seq, indicating a higher accuracy than CNV-seq. Importantly, the precise insertion position and orientation of the duplicated fragments by OGM shed light on the intact *DMD* copy, highlighting the effectiveness of OGM in prenatal risk assessment of incidentally identified CNV involving *DMD* duplication. However, practical limitations of OGM should be concerned for its poor coverage of the heterochromatin region, which awaits comprehensive analysis in combination with other methodologies. Our experience may also be instructive for application to other monogenic disorders with common etiology of SVs in, for example, *coagulation factor VIII*, *fibrillin 1*, and *neurofibromin 1* genes (Bonaglia et al, 2023; Büki et al, 2023; Fahiminiya et al, 2023).

In conclusion, we figured out the pathogenicities of Xp21.1 duplications involving 5′-terminal partial exons of *DMD* in prenatal case series using the new OGM technology, which expanded the non-deleterious variant types of *DMD*. Our findings emphasize the importance of precise structural evaluation using the OGM technology in prenatal diagnosis of dystrophinopathies and other similar monogenic disorders.

## Materials and Methods

### Patients

Four second-trimester pregnancies were enrolled in this study for an incidental prenatal finding of copy-number gains on Xp21.1 by conventional genetic technologies. All the duplications involved N-terminal partial exons of *DMD*, and were ranked as VUS according to the ACMG guideline. This study was conducted in accordance with the Helsinki Declaration of 1964 and its later amendments, and approved by the Ethics Committee of Shengjing Hospital of China Medical University (2021PS523K). Written informed consent was obtained for clinical data collection and genetic testing.

### WES analysis

Genomic DNA was extracted from amniotic fluid or peripheral blood samples using Genomic DNA Extraction Kit (QIAGEN). WES was performed using biotinylated capture probes (MyGenostics), and sequenced on the Illumina HiSeq 4000 platform (San Diego, CA, USA) with 50× coverage. The raw data were filtered and mapped to the human reference genome GRCh38. Variants were further annotated by ANNOVAR software (http://annovar.openbioinformatics.org/en/latest/), associated with multiple databases (1000 Genome, ESP6500, dbSNP, ExAC, HGMD, and Inhouse), and predicted by SIFT, PolyPhen-2, MutationTaster, and GERP++. Copy-number variation information was obtained using CNV kit (https://cnvkit.readthedocs.io/en/stable/) software.

### CNV-seq analysis

CNV-seq was performed on the Illumina NextSeq 500 platform (San Diego, CA, USA) with a depth of 0.1×. Sequences were aligned and analyzed with the human reference genome GRCh38 using the Burrows and Wheeler algorithm. Mapped reads were allocated progressively to the chromosomes, and copy-number changes were evaluated by comparing bin counts between all test samples run in the same flow cell. Several public databases including DGV, DECIPHER, OMIM, ClinGen, UCSC, and PubMed, were used to interpret the results.

### MLPA analysis

The Xp21.1 duplications involving *DMD* were confirmed in the family members using the SALSA MLPA KIT P034 and P035 probemix (MRCHolland) according to the manufacturer’s instructions. Briefly, the denatured DNA samples were hybridized with the probes at 60°C for 16 h. After ligation at 54°C for 15 min, the probes were amplified and separated by capillary electrophoresis using ABI 3730 Genetic Analyzer (Applied Biosystems). The data were analyzed using Coffalyser.Net software (MRCHolland).

### OGM analysis

High molecular weight DNA was extracted from peripheral blood leukocytes using Bionano Prep SP Blood and Cell Culture DNA Isolation Kit (80030; Bionano Genomics). The quantified DNA was labeled and backbone-stained according to the manufacturer’s guidelines. The labeled DNA was loaded onto the Saphyr chip flow cells (Bionano Genomics), and imaged sequentially across nanochannels, with each sample generating over 500 Gb of data. The data were analyzed and visualized using Bionano Solve software v3.7 and Bionano Access v1.7. The SVs were determined by aligning with the reference genome GRCh38 assembly.

## Supplementary Material

Reviewer comments

## Data Availability

The original contributions presented in the study are included in the article. Further inquiries can be directed to the corresponding author.
